# Clinical Approach to Cleft Lip and Palate with or Without Surgical Correction in Ten Brachycephalic Puppies

**DOI:** 10.3390/vetsci12050474

**Published:** 2025-05-14

**Authors:** Gleice Mendes Xavier, Keylla Helena Nobre Pacífico Pereira, Kárita da Mata Fuchs, Júlia Conseza Mendonça, Rebeca Bastos Abibe, Claudia Valéria Seullner Brandão, Maricy Apparício, Fabiana Ferreira de Souza, Matheus Gabriel Crema, Vânia Maria de Vasconcelos Machado, Maria Lucia Gomes Lourenço

**Affiliations:** 1 Department of Veterinary Clinic, School of Veterinary Medicine and Animal Science, São Paulo State University (UNESP), Botucatu 18618-681, Brazil; gleice.xavier@unesp.br (G.M.X.); keylla.pacifico@unesp.br (K.H.N.P.P.); karita.fuchs@unesp.br (K.d.M.F.); julia.cosenza@unesp.br (J.C.M.); 2 Department of Veterinary Medicine, Federal University of Alagoas (UFAL), Viçosa 57072-900, Brazil; 3 Department of Veterinary Surgery and Animal Reproduction, School of Veterinary Medicine and Animal Science, São Paulo State University (UNESP), Botucatu 18618-681, Brazil; rebeca.abibe@unesp.br (R.B.A.); valeria.brandao@unesp.br (C.V.S.B.); maricy.apparicio@unesp.br (M.A.); fabiana.f.souza@unesp.br (F.F.d.S.); matheus.crema@unesp.br (M.G.C.); vania.mv.machado@unesp.br (V.M.d.V.M.)

**Keywords:** congenital anomaly, palatoschisis, neonates, surgical palate repair, canine

## Abstract

Congenital defects are among the leading causes of neonatal mortality in small animals. Among them, cleft palate is one of the most commonly found malformations in dogs. Appropriate clinical management and surgical correction are determining factors for the survival of puppies affected by this alteration. Thus, the objective of this study is to describe 10 clinical cases of successful clinical management of cleft palate, highlighting the therapeutic strategies used, the results obtained, and the evolution of patients after treatment. Ten neonatal dogs of three breeds were treated by the Small Animal Reproduction Service of FMVZ, Unesp—Botucatu: French Bulldog (6), Pug (3), and American Bully (1) presented with cleft palate at birth or during the first week of age. Patients underwent clinical and nutritional management, in addition to immunological support. At four months of age, the patients underwent medially positioned flap repair. The animals were fed via an esophageal tube for 10 days after surgery. The clinical management proved to be effective since, throughout the follow-up, the animals showed good development and did not present complications arising from the cleft palate, and the surgical procedures were successful, restoring the quality of life of the animals.

## Simple Summary

Cleft palatine is a congenital disease that consists of a malformation of the hard and soft labia and palate (roof of the oral cavity). It can be acquired or hereditary (genetic) and affects puppies early in their lives. Treatment involves a corrective surgical procedure to repair the cleft area to ensure that food ingested by the puppy does not reach the airway. Until the puppy can undergo surgery (due to its low weight and the difficulty in obtaining good surgical access), feeding via an orogastric tube is necessary to prevent food from entering the airway, thus preventing the incidence of aspiration pneumonia in these animals. In addition, general supportive care is recommended until the puppy is of the appropriate age and body weight for the procedure. The objective of this study was to describe 10 successful cases of palatine clefts in newborn puppies of different breeds and the clinical management carried out until the congenital defect could be surgically corrected. Clinical management is very important for the puppies to survive until surgical correction of the cleft is performed.

## 1. Introduction

Cleft palate in dogs is a congenital malformation that occurs due to failure of fusion of the palatine plates during embryonic development, resulting in an opening in the secondary palate, a structure responsible for forming the roof of the oral cavity and separating this cavity from the nasal cavity. The process of palate development in dogs follows a precise sequence of morphological events during gestation, similar to that observed in humans and other mammals. In dogs, the palate closes between the middle and final thirds of gestation, with complete fusion occurring around day 44 [1,2,3,4].

Genetic factors such as the concentration of specific alleles resulting from consanguineous crosses, such as the ADAMTS20 and DLX6 genes, are some genes shared between dogs and humans that increase the incidence of cleft palate in dogs [4,5], and/or dysfunctions of the regulators and migrators of mesoderm and neural crest cells. Similarly, teratogenic and environmental factors such as the use of certain drugs (for example, tetracyclines, aspirin, and corticosteroids), hypervitaminosis A, folate deficiency, and infectious agents (for example Canine Distemper virus (CDV)) may contribute to the appearance of this condition in dogs, which is more common in brachycephalic dogs [1,2,3,5,6].

The size and depth of the opening tends to vary from small fissures to large openings that affect general health of the neonate [4]. In more severe cases, newborns present challenges that can influence their vitality, such as suckling and breathing difficulty, in addition to an increased risk of respiratory infections due to abnormal communication between the oral and nasal cavities, making these animals much more likely to develop aspiration pneumonia than newborns who do not have any opening in the palate region [7].

Congenital defects are among the main causes of neonatal mortality in small animals. Among them, cleft palate is the most common malformation in dogs, with an incidence of 2.8%. Appropriate clinical management and surgical correction are determining factors for the vitality of neonates affected by this alteration [8]. Conservative treatment, which involves clinical and nutritional care, prevents the appearance of systemic complications, favoring the adequate development of the newborn, facilitating surgical access and, consequently, promoting good postoperative results. Thus, this study describes 10 clinical cases of successful clinical management of cleft palate, highlighting the therapeutic strategies used, the results obtained, and the evolution of patients after treatment.

### 1.1. Palate Morphogenesis

Craniofacial development is a sequence of regulated events that begins with the participation of the frontonasal prominence and pharyngeal arches [4,9,10]. In this process, cell multiplication, migration and differentiation, tissue fusion, and apoptosis occur, and these processes are dependent on transcription factors regulated through gene expression [2,11].

In the embryo, the stomodeum or primitive mouth is observed as a depression in the ectoderm surrounded by mesenchymal structures, where there is proliferation of neural crest cells, which are important for the development of craniofacial structures [12]. Among the components that surround the stomodeum is the frontonasal prominence, in which a pair of lateral and medial nasal processes originate; the fusion between these portions of each process delimits the nasal cavity in formation. Later, the medial processes elongate and project between the maxillary prominences, joining them and transforming into the primary palate [13].

**Figure 1:**
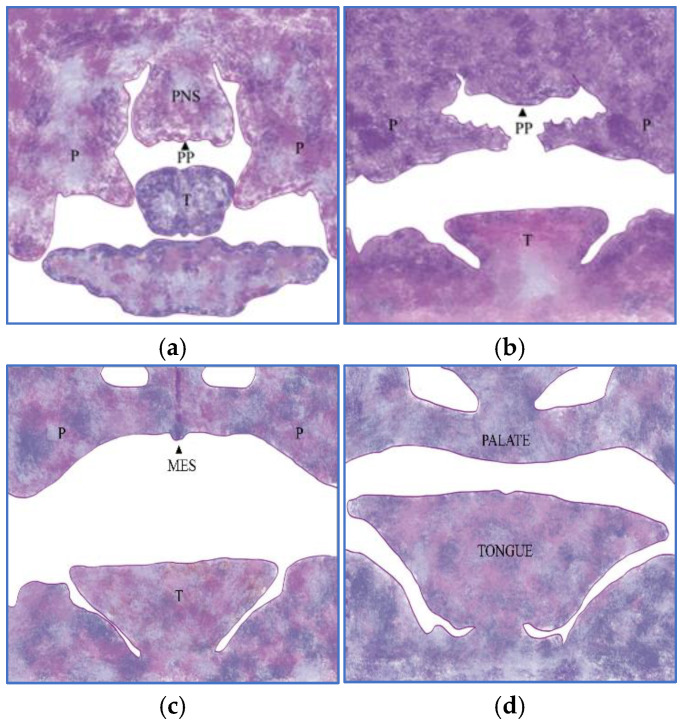
Embryonic development of the palate (schematic drawing). (**a**): Developmental phase of the palatine shelves, (**b**): Elevation phase, (**c**): Adhesion phase, (**d**): Fusion phase. PNS—Primitive nasal septum; PP—Primitive palate; P—Palatine shelfs; T—Tongue; MES—Medline epithelial suture.

Studies have shown that palate development can be divided into four stages: development, elevation, adhesion, and fusion [1,4]. First, the palatine shelves develop (Figure 1a), projecting vertically laterally to the tongue. The shelves are subsequently elevated and projected horizontally over the tongue (Figure 1b), where both shelves meet and adhere to form the medial epithelial suture (MES) (Figure 1c), followed by fusion of the shelves and disappearance (Figure 1d).

Studies have revealed that the formation of the palate in dogs can occur between the 25th and 39th days of gestation, with the fusion of the shelves occurring between the 33rd and 39th days of gestation, with complete fusion occurring around the 44th day of gestation, considering day 0 as the day of mating and/or artificial insemination [4,14,15,16,17].

### 1.2. Etiology

Cleft palates and/or fissures in dogs can be caused by environmental or genetic factors or a combination of these factors [18,19]. In humans, approximately 25–30% of cleft palate cases can be attributed to genetic factors, approximately 20 genes are involved in the development of the palate in humans, and a failure in their expression and/or action can result in structural defects [1,20,21].

Some breeds, such as English Bulldog, French Bulldog, Boston Terrier, Boxer, Cavalier King Spaniel, Pug, Labrador Retriever, and Schnauzer [1,20,21], have a genetic predisposition to a palliative cleft, conferring a hereditary character for the appearance of these alterations. Studies indicate that inheritance can be recessive monogenic or multifactorial, and the breed and environmental factors can contribute to the development of these diseases [12,13,14,15,16,17,18,19,20,21,22,23,24,25,26,27,28,29].

Mutations in the DLX6 and ADAMTS20 genes are associated with the development of cleft palate and other abnormalities in dogs. The LINE-1 insertion in the DLX6 gene reduces its expression, compromising the development of the maxilla and mandible, which contributes to the incidence of the malformation. ADAMTS20, in turn, undergoes a deletion of two nucleotides, resulting in the truncation of a protein essential for the formation of the palate [11,12]. To avoid autosomal recessive clefts, it is important not to cross heterozygous individuals with each other, avoid consanguineous unions in case of family history, and avoid crossing dogs with any relationship for multifactorial clefts.

Nutritional factors can also contribute to the incidence of congenital anomalies; in this context, maternal nutrition plays a crucial role in embryonic development. One example is folic acid deficiency, which can contribute to the appearance of malformations in the neural tube and palate in several species, including dogs. However, the effectiveness of prepartum supplementation is still being addressed, since it is possible to observe the incidence of palatoschisis in puppies of females supplemented with folic acid during embryogenesis [6,30,31,32]. Hypervitaminosis A (a precursor of retinoic acid) can induce saturation and/or inactivation of intracellular receptors or membranes, interfering with cell signaling mediated by retinoic acid, and resulting in hypoplasia of the palatine shelves, causing clefts or other craniofacial anomalies [33,34].

Exposure to teratogens has been described as a causative agent of craniofacial anomalies. Teratogens are substances that interfere with fetal development and may predispose dogs to cleft palate [17,35,36,37]. The administration of corticosteroids during the initial third of gestation may influence the appearance of cleft palate in canine fetuses. This process occurs because these corticosteroids interfere with the proliferation and differentiation of embryonic tissues [35]. Maternal viral infections such as canine distemper virus (CDV) can impair the development of fetal tissues, generating craniofacial anomalies such as cleft palate in canine fetuses [37]. Exposure to pesticides, chemical agents, and other drugs can lead to cleft palate, as they affect cell migration and palliative fusion [36].

The occurrence of some hormonal and metabolic disorders, whether maternal or intrauterine, can affect fetal metabolism, impairing the cellular differentiation of tissues during pathogenesis [38]. Fetal hypoxia can occur due to placental problems and maternal hemodynamic disorders, and the lack of oxygenation compromises cell proliferation due to a reduction in adenosine triphosphate (ATP) in embryonic cells, resulting in a failure of the cell migration and adhesion necessary for complete fusion of the palate [39,40].

### 1.3. Types of Palatoschisis

**Figure 2:**
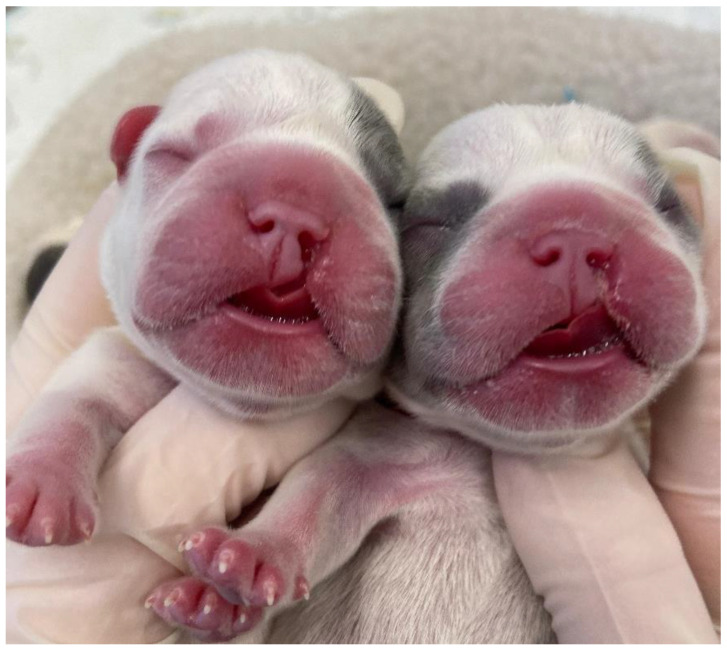
Cleft lips of two neonate dogs.

**Figure 3:**
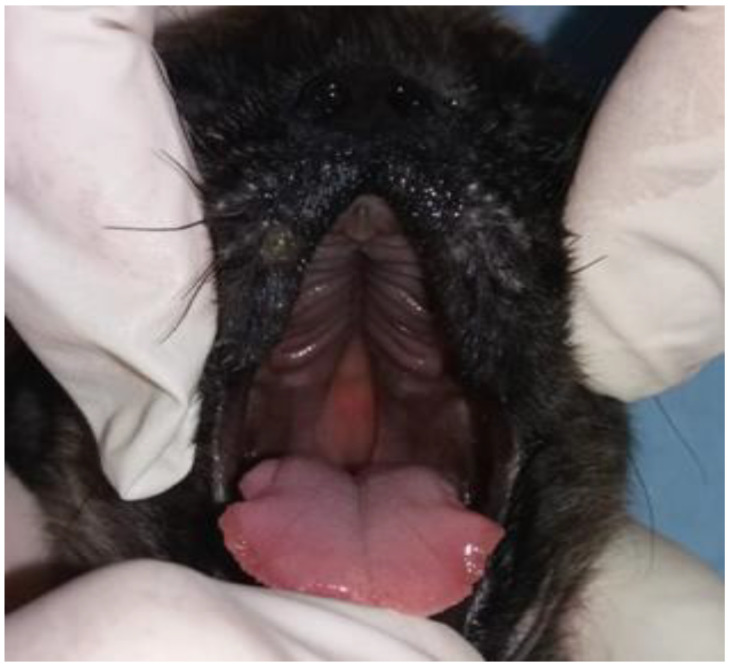
Complete cleft palate in a neonate dog.

In the literature, terms such as congenital cleft palate, cleft palate, or palatoschisis can be found. The first is the result of failure to close the primary and/or secondary cleft palate. In these cases, the anomaly is associated with defects in the upper lip (cleft lip) (Figure 2) and upper jaw (gnathoschisis) [41]. The second is related to defects in the palate during the development of the secondary palate. These can be classified according to the degree of involvement of the structures. Clefts that involve the hard and soft palates are considered complete clefts (Figure 3), whereas incomplete clefts involve only the soft palate [42,43].

Thus, the clinical signs of this anomaly are nonspecific. When not diagnosed, neonates with cleft palate may present with milk coming out of the nostrils, choking, weak sucking, weight loss, growth impedance, and neonatal triad (hypoglycemia, hypothermia, and dehydration) [8,44]. The diagnosis is made by inspecting the oral cavity and confirming the cleft. The treatment of choice is surgical correction known as flap repair, which is indicated to be performed from three months of age, an age that provides the best surgical access and reduces the incidence of recurrence. Importantly, until the time of surgical intervention, patients undergo clinical and nutritional management with feeding and water supplied via tubes [44,45].

## 2. Case Series Presentation

Ten newborn dogs of three breeds, French Bulldog (*n* = 6), Pug (*n* = 3), and American Bully (*n* = 1), were treated by the Small Animal Reproduction Service of FMVZ, Unesp—Botucatu. They were diagnosed with cleft palate at birth or during the second week of age (Table 1). Of these, two puppies arrived at the clinic with clinical signs of aspiration pneumonia and three had signs of the neonatal triad (hypothermia, hypoglycemia, and dehydration). The other five patients were diagnosed at birth and did not present with systemic alterations resulting from the anomaly.

**Figure 4:**
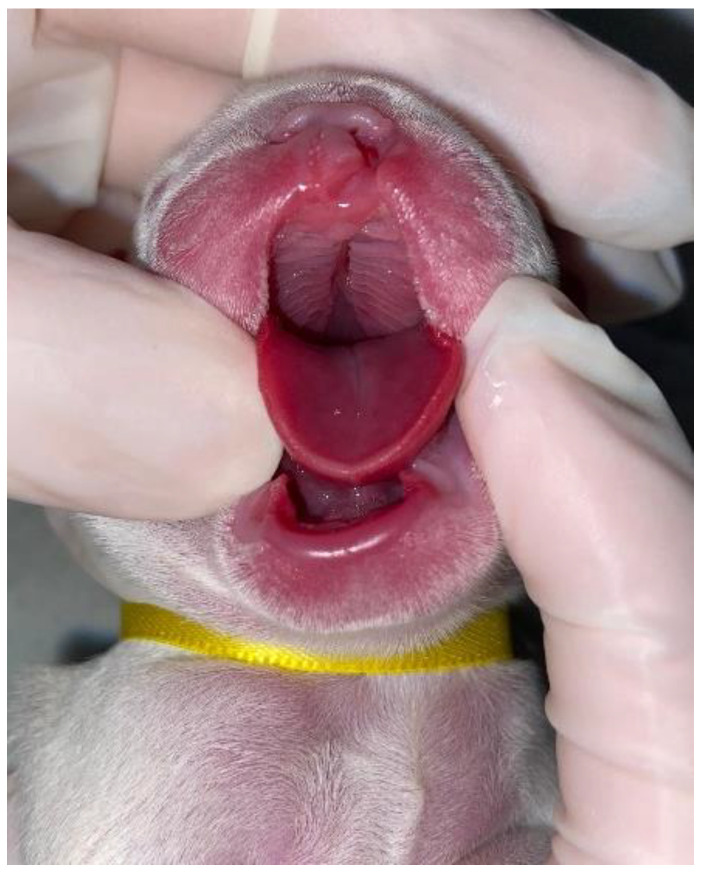
Cleft palate associated with unilateral cleft lip in a neonate dog.

On physical examination, nine neonates were male and had a complete cleft palate, with involvement of the hard and soft palate, and one newborn female (French Bulldog) also had a unilateral cleft lip associated with a complete cleft palate (Figure 4). Two pug puppies presented crepitation in the lung fields, so a radiographic examination was requested in which bronchoalveolar opacification was observed, suggestive of aspiration pneumonia. Three puppies had associated malformations, such as pectus excavatum, hydrocephalus, and cleft lip.

**Figure 5:**
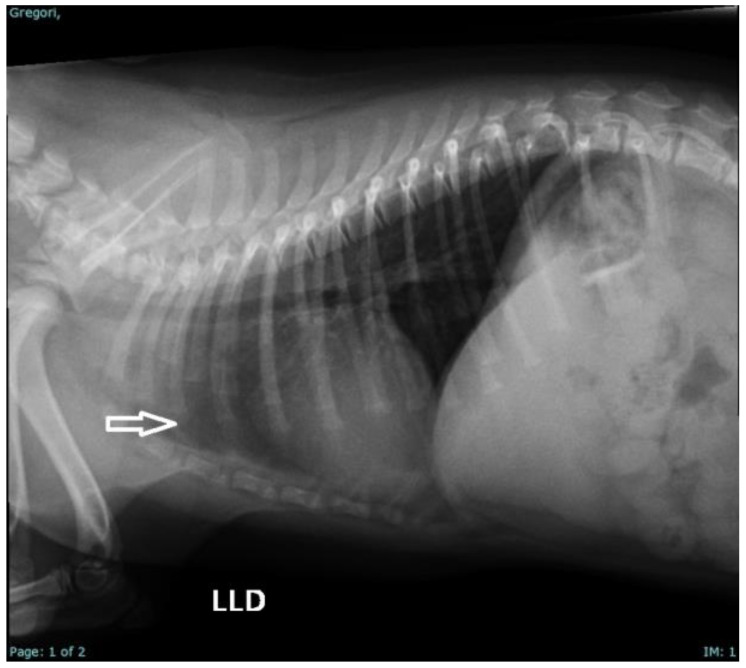
Radiographic examination of a French bulldog puppy demonstrating radiopacity in the cranial mediastinum region due to aspiration pneumonia.

During the hematological examination, the neonates who presented with aspiration pneumonia (Figure 5) presented leukocytosis due to neutrophilia with a shift to the left (>20,000 mg/dL), and only one neonate presented associated monocytosis (1800 mg/dL). Treatment was initiated with the provision of oxygen therapy and nebulization with saline solution (TID), N-acetylcysteine (3 mg/kg/VO/TID, for 5 days) and amoxicillin + clavulanate (20 mg/kg, VO, BID, for 7 days) was prescribed for home use. The other neonates did not present hematological alterations that indicated any systemic inflammatory/infectious process.

Thus, neonates who presented signs of triad syndrome were stabilized, in which 12.5% glucose (0.5 mL/100 g) was administered, fluid therapy with lactated Ringer’s solution (2 to 4 mL/100 g of weight bolus + maintenance of continuous infusion (0.5 mL/100 g/h)) and provision of a heat source to reestablish body temperature. The puppies that presented congenital alterations associated with cleft palate were stabilized, with the protocol indicated for each alteration, as described in Table 1.

**Figure 6:**
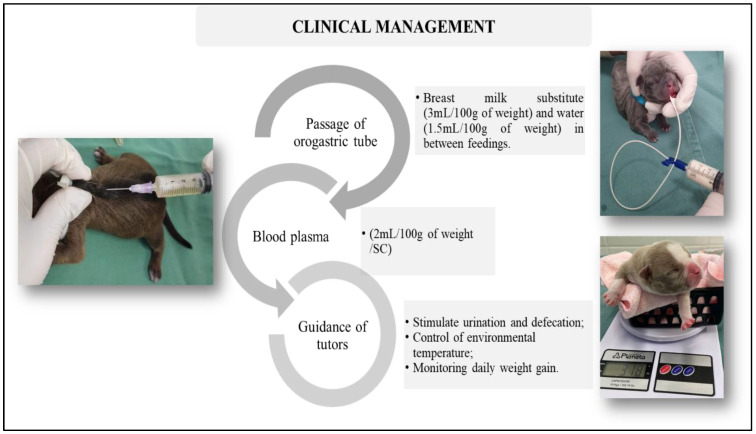
Diagram of the clinical management of cleft palate in neonatal dogs.

After stabilization of the clinical conditions, the patients underwent nutritional management via an orogastric tube (urethral tube no. 6), breast milk substitute (3 mL/100 g of weight), and water between feedings (1.5 mL/100 g of weight). As a source of passive immunity, immunological management was performed with the administration of blood plasma (2 mL/100 g of weight), subcutaneously in a single dose at the time of the first treatment. The guardians were instructed to stimulate urination and defecation with damp cotton after feeding, environmental heating, and monitoring of daily weight gain (Figure 6).

Clinical management: Feeding via orogastric tube; administration of blood plasma (2 mL/100 g of weight); stimulation of urination and defecation and weighing.

**Figure 7:**
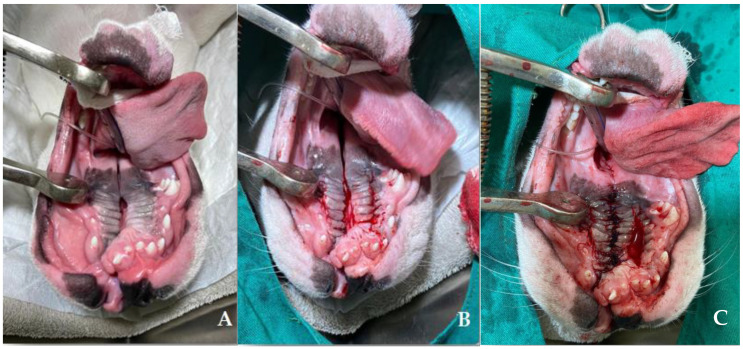
Medially positioned flap repair procedure in a French bulldog puppy. (**A**): Patient in dorsal decubitus, in which medial incisions were made parallel to the maxillary dental arches. (**B**): Divulsion of the hard palate mucosa in the maxillary region for subsequent advancement flap, executed in the lateral-medial direction along the entire length of the mucosa. (**C**): The bundles were joined with simple stitches, and 2-0 absorbable monofilament synthetic sutures. In the soft palate, tissue debridement and continuous double-layer suturing with 3-0 absorbable synthetic sutures were performed.

**Figure 8:**
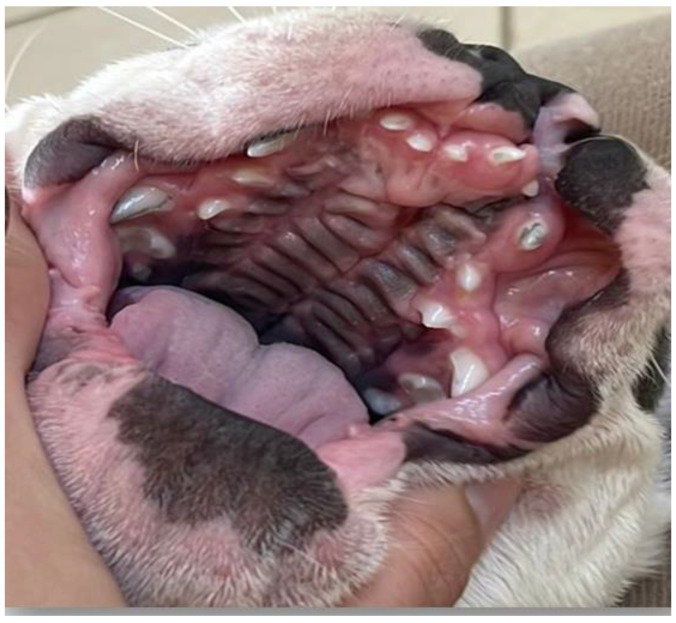
Post-surgical staphylorrhaphy in a French bulldog puppy.

**Figure 9:**
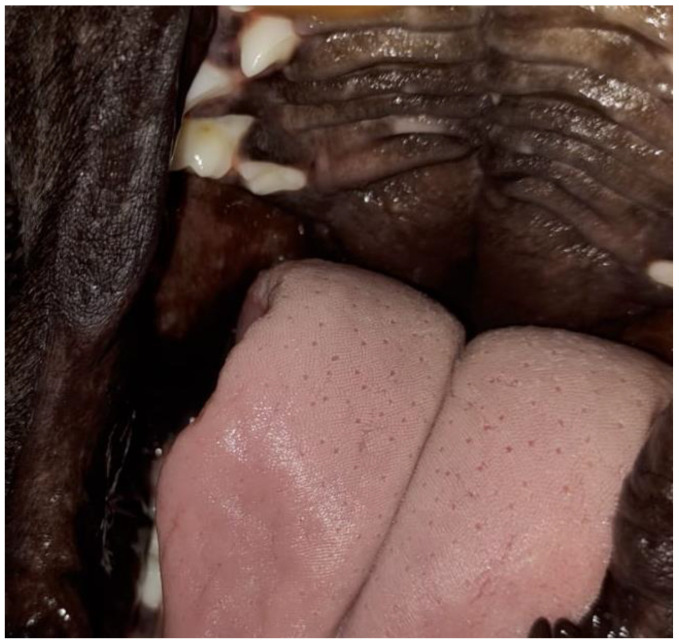
Spontaneous closure of a cleft palate in a French bulldog puppy.

At four months of age, nine of ten the patients underwent medially positioned flap repair, following the techniques described by [46] (Figure 7). The animals were fed via an esophageal tube for 10 days after surgery. Follow-ups were performed to assess the healing process and to introduce a soft diet 10 days after the surgical procedure (Figure 8). A puppy (French bulldog) presented spontaneous closure of the cleft palate (Figure 9). Only three patients required surgical reintervention (following the same techniques described previously) one month after the first procedure. All the pups were monitored three months after the procedure, and no complications were observed due to clinical and/or surgical management.

## 3. Discussion

The cleft palate in dogs is considered a multifactorial congenital anomaly; genetic and environmental factors may be correlated with the incidence of this alteration. Studies suggest that the alteration and/or interaction of genes involved in the development of the palate is the main cause of this anomaly in dogs [12,29]. In the animals in the present study, it was not possible to identify the cause of cleft palate; however, the genetic hypothesis is the most accepted because all the animals in the study were brachycephalic and all the pregnant females had good nutritional scores. The racial predisposition observed in brachycephalic breeds reinforces the influence of genetic selection on the incidence of this condition [8,23,27].

The clinical manifestations of cleft palate in dogs may include symptoms such as difficulty feeding, regurgitation, aspiration pneumonia, coughing, frequent respiratory infections, insufficient growth, and even dental problems [7]. In the patients in the present study, it was possible to observe growth deficit; some had milk coming out of the nostrils and those who had aspiration pneumonia experienced respiratory distress. The diagnosis is made on the basis of the patient’s clinical history and specific physical examination [7,8,44].

The presence of aspiration pneumonia in two of the puppies corroborates what has been described in the literature as a complication of cleft palate in neonates [44,46,47]. The clinical signs of pulmonary crepitus and the radiographic changes observed are indicative of pulmonary inflammation secondary to aspiration, requiring immediate therapeutic intervention [16].

Therefore, the first time the puppies were seen, their respiratory condition was stabilized, and clinical management of the cleft palate was implemented, with the aim of preventing milk aspiration and recurrence of the alteration. The puppies that presented clinical signs of the triad were stabilized, following the protocols recommended for the species, with the administration of 12.5% glucose, fluid therapy with lactated Ringer’s solution, and heat therapy, aimed at the recovery of these patients [44,48].

Nutritional management through orogastric tube feeding is essential to ensure adequate nutrition for newborns and prevent milk aspiration, allowing their proper development. Immune supplementation with blood plasma aims to provide passive immunity, since these newborns may have limited absorption of immunoglobulins due to inadequate sucking, as well as limited absorption of these immunoglobulins due to intestinal development [49,50].

A puppy (French bulldog) presented spontaneous closure of the cleft palate (Figure 9), which is considered a rare phenomenon and is rarely documented in the literature. The justification for this event is that neonates have active tissue growth factors, so in smaller clefts, fibroblast proliferation and migration to the lesion focus may occur, promoting collagen synthesis and filling the defect [51,52]. Appropriate clinical management appears to be a precursor for cleft closure, since orogastric feeding prevents local inflammation, reducing the incidence of secondary infections, and promoting tissue regeneration [44,53].

The protocols established for the treatment of congenital alterations associated with cleft palate, described in Table 1, were effective in stabilizing and/or treating the alterations. The management of the box, with constant changes in position, has already been described as an effective technique for the treatment of pectus exacavatum in canine neonates [54]. In the present study, correction of the alteration after six days of treatment was possible. Just as the treatments based on furosemide, corticosteroids, and omeprazole demonstrated effectiveness for the supportive treatment of hydrocephalus, during the entire clinical follow-up, the patient did not present clinical symptoms correlated with the alteration.

**Figure 10:**
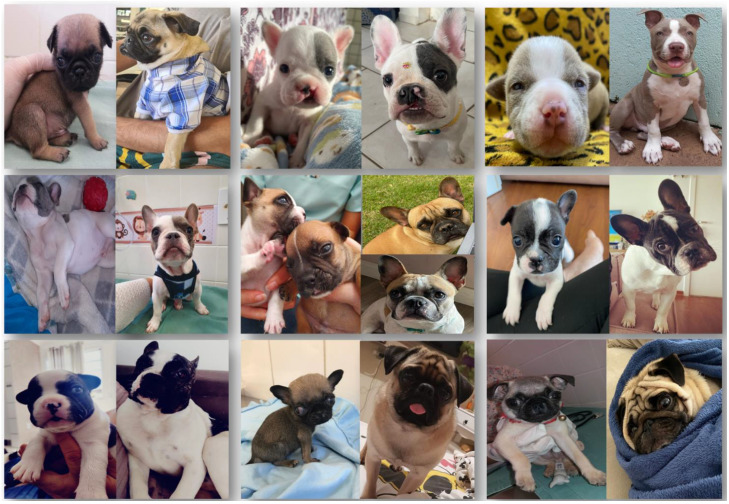
Before and after clinical and surgical management of 10 neonates with cleft palate.

Surgical correction of cleft palate via the surgical flap technique is the definitive treatment and should be performed after three to four months of age, as described by [46], with high success rates [44,45]. Esophageal tube feeding in the immediate postoperative period is essential to avoid trauma in the surgical region, favoring healing, since it is possible to reduce the chances of contamination and consequently dehiscence of stitches resulting from secondary infections [55]. In the cases of the present report, successful management contributed to the clinical stability of the neonates and, consequently, to the surgical intervention, highlighting the importance of a multidisciplinary and individualized approach for these patients, contributing to their healthy growth (Figure 10).

## 4. Conclusions

Clinical management and surgical procedures have proven to be effective, resulting in a significant improvement in the quality of life of the animals. A multidisciplinary approach, which included clinical stabilization, nutritional support, and surgical interventions, was essential for the successful management of neonates with cleft palate. During the four months of follow-up, the patients did not present complications resulting from the malformation, allowing adequate development and providing good surgical access, which contributed to the success of the surgeries. Periodic clinical monitoring is crucial to ensure a satisfactory postoperative evolution and maintain the quality of life of these patients. The recognition and adequate management of this condition are essential for the survival of the neonates, with early clinical intervention being a determining factor in the prognosis. Regarding the predisposing factors for the development of cleft palate, the present study was unable to identify a specific factor, although genetic predisposition is the most acceptable cause, since all the animals studied were brachiocephalic. Additionally, the present study found that the incidence was higher in male neonates.

## Figures and Tables

**Table 1:** Identification of neonate dogs with descriptions of clinical signs and treatments instituted.

Neonate	Breed	Gender	Diagnosis	Clinical Signs	Treatment
1	American Bully	Male	At birth	Weak sucking and pectus excavatum	Clinical management + box management
2	French Bulldog	Male	At birth	Weak sucking	Clinical management
3	French Bulldog	Male	At birth	Weak sucking	Clinical management
4	French Bulldog	Male	At birth	Weak sucking	Clinical management
5	French Bulldog	Male	At birth	Weak sucking *	Clinical management
6	French Bulldog	Female	At birth	Cleft lip and weak sucking	Clinical management
7	French Bulldog	Male	At birth	Weak sucking	Clinical management
8	Pug	Male	At 3 days of life	Gagging, milk coming out of the nose, weak sucking	Clinical management
9	Pug	Male	At 2 days of life	Sepsis, dyspnea, pulmonary crackles, neonatal triad, and weak sucking	Clinical management + Amoxicillin + clavulanate (20 mg/kg/BID/7 days); N-acetylcysteine (3 mg/kg/PO/TID, for 5 days) and Nebulization (TID) [44]
10	Pug	Male	At 14 days	Sepsis, dyspnea, pulmonary crackles, neonatal triad, weak sucking, and hydrocephalus	Clinical management; Amoxicillin + clavulanate (20 mg/kg/BID/7 days); N-acetylcysteine (3 mg/kg/PO/TID, for 5 days); Nebulization (TID); Prednisolone (0.5 mg/kg/PO/BID/7 days); Omeprazole (1 mg/kg/PO/SID); Furosemide (2 mg/kg/BID/2 days) [44]

* Spontaneous closure of the cleft palate.

## Data Availability

All data generated or analyzed during this study are included in this published article.
